# Supporting Physical Activity and Quality of Life in Older Adults with Coronary Heart Disease: Development of a Web-Based Nursing Intervention

**DOI:** 10.1177/08445621261452849

**Published:** 2026-05-26

**Authors:** Audrey Lavoie, Véronique Dubé

**Affiliations:** 1 Faculty of Nursing, 63678 Université de Montréal, Montreal, QC, Canada; 2 Université de Montréal Marguerite-d’Youville Research Chair on Humanistic Nursing interventions, Montreal, QC, Canada; 3 Research center, Centre Hospitalier de l'Université de Montréal, Montreal, QC, Canada

**Keywords:** coronary heart disease, web-based nursing intervention, older adults, physical activity, quality of life

## Abstract

**Background:**

Coronary heart disease (CHD) remains a leading cause of morbidity and mortality worldwide. For older adults, sustained engagement in physical activity after coronary revascularization is essential for secondary prevention and is strongly associated with improved quality of life. However, participation in traditional cardiac rehabilitation programs remains low in this population, highlighting the need for accessible, age-tailored alternatives.

**Objective:**

To describe the development of *Changeons ensemble*, a French-language web-based nursing intervention designed to promote physical activity and quality of life among older adults with CHD.

**Methodology:**

The intervention was developed by a multidisciplinary participatory planning group following the Intervention Mapping framework. A comprehensive needs assessment was conducted through a literature review and semi-structured interviews with older adults (n = 10). The subsequent steps included objective setting and intervention design guided by the Information–Motivation–Behavioral Skills model.

**Results:**

The needs assessment identified key needs in information, motivation, and self-efficacy and highlighted the importance of individualized professional support. *Changeons ensemble* was designed as a seven-session web-based intervention integrating educational content, reflexive activities, individualized written nursing feedback, action planning, self-monitoring through an electronic diary, a forum, and case stories. The intervention emphasizes flexibility and sensitivity to older adults’ physical and emotional challenges following coronary revascularization.

**Conclusion:**

This study presents a systematically developed, theory-informed, web-based intervention that directly addresses the specific needs of older adult with CHD. *Changeons ensemble* offers a promising approach for supporting sustained physical activity and enhancing in quality of life in a population that remains underserved by conventional cardiac rehabilitation models.

**Trial Registration:**

ClinicalTrials.gov NCT06197347; https://clinicaltrials.gov/study/NCT06197347

## Background

Cardiovascular diseases, including coronary heart disease (CHD), remain one of the leading causes of death worldwide and are associated with significant health consequences in older adults, such as arrhythmias, heart failure, and stroke (World Health Organization, 2017). While coronary revascularization procedures (e.g., coronary artery bypass grafting and percutaneous coronary intervention) are essential, the long-term management of CHD relies heavily on secondary prevention strategies aimed at improving lifestyle habits (e.g., adopting a healthy diet, smoking cessation, stress management, and regular physical activity) (Kotseva et al., 2019). Specifically, adopting or maintaining physical activity is crucial to prevent complications and reduce mortality among older adults following coronary revascularization (Anderson et al., 2016; Lahtinen et al., 2018; Lopes et al., 2020). In addition to its cardioprotective effects, physical activity is also associated with improvements in quality of life (Gianluca et al., 2020), which is a key concern and priority for many older adults post-revascularization (Jepma et al., 2021).

Secondary prevention interventions, such as cardiac rehabilitation programs, are recognized as effective strategies for promoting healthy lifestyle habits among individuals with CHD (Ambrosetti et al., 2020). These programs typically include multidisciplinary follow-up, structured group exercise sessions, and psychological support (Ambrosetti et al., 2020). Despite their well-established benefits, cardiac rehabilitation programs remain underutilized by older adults (Resurrección et al., 2019). Older adults may have difficulty accessing these center-based programs, or may be excluded due to frailty, physical limitations or the use of walking aids (Sumner et al., 2016). Additionally, healthcare professionals may be less likely to refer older patients to these programs due to their age, comorbidities, and perceived lower ability to participate (Astley et al., 2020), even though they could significantly benefit from them (Alfaraidhy et al., 2022; Lutz & Forman, 2022).

Furthermore, most existing rehabilitation programs are designed for individuals of all ages living with CHD, without specific adaptations for older adults. Yet, several authors have emphasized the importance of developing age-tailored interventions that address the unique challenges and needs of this population (Allemann & Poli, 2020; Lutz & Forman, 2022). Older adults represent a highly heterogeneous group with diverse characteristics and life contexts (e.g., health status, frailty, functional capacity, changes in social roles), which may influence their secondary prevention needs (Alfaraidhy et al., 2022). However, these needs remain underexplored, particularly in relation to behavioral change. In particular, several behavioral determinants may act as barriers to the adoption and maintenance of healthy lifestyle habits in this population. Older adults with CHD can experience challenges such as limited motivation to change lifestyle habits, reduced self-efficacy associated with physical limitations, and an insufficient awareness of the benefits of secondary prevention (Cleary et al., 2015; Jepma et al., 2021). Given these challenges, healthcare professionals, particularly nurses, may play a key role in supporting behavior change. Given their strong relational skills, which are highly valued by older adults (Beishuizen et al., 2019; Lavoie, 2018; Ligthart et al., 2015), as well as their well-established role in managing chronic diseases, including CHD (Yousefi et al., 2019), nurses may help enhance motivation, strengthen self-efficacy, and increase awareness of the benefits of lifestyle changes, thereby supporting the adoption and maintenance of health behaviors. In this context, alternative interventions to cardiac rehabilitation programs may help address existing barriers to access and support behavior change among older adults.

Cardiac rehabilitation can be delivered through various modalities (e.g., in-center, home-based, web-based, or technology-assisted) (Babu et al., 2020). Given the access barriers to in-center programs and the lack of human and financial resources required for in-person home-based interventions (Lima de Melo Ghisi et al., 2018), web-based interventions appear to be a promising alternative. Several authors suggest that web-based interventions can promote physical activity and enhance quality of life among individuals with CHD (Platz et al., 2023; Su & Yu, 2021). However, despite this promise, to our knowledge, no web-based interventions have been specifically designed to promote physical activity among older adults living with CHD. This lack of tailored interventions represents a significant gap in cardiac rehabilitation, limiting the potential benefits of web-based solutions for this growing population. Drawing from broader research, for such interventions to be effective, they would need to be tailored to older adults’ specific needs (Ligthart et al., 2015; Richard et al., 2019), include professional support (Pols, 2017; van Middelaar et al., 2018), and be grounded in behavioral change theory (Koulouvari et al., 2025). Therefore, the role of professional support in web-based interventions for older adults remains largely underexplored (Brørs et al., 2019; Lavoie & Dubé, 2022). Theoretical models provide a systematic framework for understanding why individuals adopt or fail to adopt health behaviors. They help identify modifiable factors (e.g., knowledge, motivation, self-efficacy) and guide the selection of evidence-based strategies to influence them (Bartholomew et al., 2016; Kok et al., 2016). Consistent with this, interventions developed using behavioral theory and targeting key determinants of health behavior are more likely to achieve meaningful and sustained behavior change. Therefore, it is critically important to develop accessible, theory-informed web-based interventions that provide individualized nursing support tailored to the specific needs of older adults, while targeting key factors influencing physical activity. To our knowledge, no such intervention currently exists, highlighting a crucial opportunity for innovation in cardiac rehabilitation.

### Aim

The aim of this article is to describe the development of *Changeons ensemble,* a French-language web-based nursing intervention designed to promote physical activity and quality of life in older adults with CHD.

### Framework for the Development of the Intervention

The Intervention Mapping (IM) framework proposes a model for developing health interventions that includes all decision-making steps (Bartholomew et al., 2016). In the present study, the use of this framework allowed for a systematic approach to developing the intervention, based on both data collected from a population and empirical data (Bartholomew et al., 2016). The IM approach aligns with the development of a third-generation intervention that prioritizes a prior needs assessment, theoretical foundations, and the active involvement of end-users to enhance uptake and sustainability (Gagnon et al., 2012). Development of the intervention followed Steps 1 through 4 of the IM framework (Bartholomew et al., 2016), as described below.

### Step 1: Needs Assessment

The first step in the IM framework involves conducting a needs assessment to understand the issues faced by the target population. This was achieved through a literature review, as well as semi-structured interviews.

#### Literature Review

A literature review was conducted to inform the needs assessment. The review initially aimed to identify studies exploring the needs of older adults living with CHD in relation to secondary prevention intervention. However, given the limited availability of such studies, the scope was broadened to include studies exploring health experiences and behavior change among this population. Relevant studies were identified through targeted searches in key databases and selected based on their relevance to the research objectives, resulting in the inclusion of five studies.

#### Sampling and Participants

Participants were recruited using convenience sampling (Polit & Beck, 2017). Inclusion criteria were: (1) being aged 65 years or older; (2) having been hospitalized within the past year following coronary artery bypass grafting or percutaneous coronary intervention; (3) reporting having increased or attempted to increase their physical activity level following hospitalization; and (4) being able to understand and speak French. These inclusion criteria were established to recruit older adults during a clinically relevant transition period within 12 months following coronary revascularization, which is considered a critical window for initiating health behavior change (Cleary et al., 2015). Participants were selected based on their lived experience with post-revascularization recovery to capture retrospective insights regarding their needs and support requirements in adopting physical activity behaviors. The exclusion criterion was the presence of a moderate to severe neurocognitive disorder, as documented in the medical record, including impairments in complex attention, executive functions, learning abilities, memory, or social cognition.

A participatory planning group was also established, by inviting professionals working in the clinical setting involved in participant recruitment to indicate their interest in contributing to the intervention development process. Interested professionals were selected based on their relevant expertise in the fields of CHD, aging, and physical activity (geriatrician, kinesiologist, physiotherapist, cardiology nurse). An older adult living with CHD was also invited to contribute to ensure the inclusion of a lived experience perspective.

The participatory planning group provided iterative feedback on each session of the intervention, with members contributing according to their area of expertise (e.g., physiotherapist input on functional limitations, geriatric expertise on age-related considerations, and nursing input on chronic disease management and patient support). The patient partner contributed throughout the intervention development process by providing experiential feedback based on their lived experience of CHD. Specifically, this input helped ensure that the intervention content was aligned with the needs and realities of individuals living with CHD, particularly in relation to recovery, daily functioning, and engagement in physical activity.

All feedback was integrated and refined by the lead researcher. This multidisciplinary group ensured that the intervention was informed by both clinical expertise and lived experience.

#### Data Collection

The interview guide included three sections: (1) health experience, including questions about the impact of CHD and physical activity (e.g., What are the impacts of CHD on your life?); (2) behavior change, including questions that explore physical activity levels, knowledge, motivation, and self-efficacy (e.g., Since your diagnosis of CHD, would you have liked to, or have you succeeded in being more active on a daily basis? I’d like you to tell me about your experience), and (3) a secondary prevention intervention, with questions that explore preferred components of the web-based intervention (e.g., What should this web-based intervention have included (e.g., content, resources, support) to help you become more active?).

#### Recruitment Procedure

Eligible participants were identified from a university hospital registry of patients hospitalized within the past year for coronary artery bypass grafting or percutaneous coronary intervention. An administrative assistant first contacted eligible individuals to obtain permission for the research team to reach them. Upon authorization, the principal investigator contacted potential participants to confirm eligibility and assess interest in participation. Interested and eligible individuals were then scheduled for either a telephone or in-person meeting, according to their preference, during which informed consent was obtained and the semi-structured interview was conducted.

#### Data Analysis

Following transcription of the interviews, they were imported into the qualitative data analysis software MAXQDA (VERBI Software, Germany). A thematic analysis was performed (Paillé & Mucchielli, 2021). From the participants’ verbatim statements, themes (i.e., labels summarizing a set of related data) and sub-themes, or decompositions of themes, were identified (e.g., theme: encouragement; sub-themes: encouragement promotes progress, encouragement validates success). Themes were then compared and grouped based on complementarity, similarity, recurrence, and divergence to form salient thematic clusters, i.e., sets of themes sharing common characteristics (e.g., sources of motivation). Relationships among these themes were further examined through the creation of thematic axes, representing significant poles that integrate salient thematic clusters (e.g., support in facing behavioral change). These axes and clusters were hierarchically organized, resulting in a thematic tree diagram illustrating the structure of the analysis (Paillé & Mucchielli, 2021). Special attention was paid to identifying themes directly related to the study's objectives, research questions, and the IMB concepts, ensuring low inference levels and close alignment with the participants’ statements (Paillé & Mucchielli, 2021). Additionally, journal entries were analyzed using a thematic approach, identifying themes and sub-themes to complement the interview analysis. Data collection and analysis were conducted iteratively, and thematic saturation was considered during the analytic process.

### Step 2: Identification of Intervention Goals

The second step aimed to determine the intended outcomes of the intervention for older adults with CHD. Based on the needs assessment, a primary goal was established: to promote physical activity and improve quality of life. This goal was then broken down into specific performance objectives aligned with the behavior change factors that needed to be addressed to achieve the desired outcomes.

### Step 3: Intervention Design

Step 3 involved determining the structure and components of the intervention, including the themes, scope, theories and behavior change techniques. To determine the design of the intervention, we drew on the results of the needs analysis as well as literature on existing web-based interventions for older adults (Lavoie & Dubé, 2022). Relevant behavior change theories were then analyzed and selected based on their constructs and applicability to the factors influencing behavior change identified in Step 1. These factors were then linked to behavior change techniques, drawing from the taxonomy by Michie et al. (2013) and the study by Carey et al. (2019). Carey et al. (2019) identified connections between these techniques and behavior change theories to ensure the intervention could lead to meaningful changes.

### Step 4: Intervention Production

In Step 4, the content of the intervention was developed iteratively, in collaboration with the participatory planning team. Each expert provided feedback on the intervention's content based on their expertise. The physiotherapist makes certain that the content was consistent with post-revascularization recommendations and the potential physical limitations of the target population. The kinesiologist ensured that the proposed exercises were safe. The geriatrician validated that the intervention was consistent with an approach to care adapted for older adults. The cardiology nurse provided expertise related to CHD and the symptoms that may be experienced after coronary revascularization. The first author incorporated the suggested modifications and returned revised versions to the team for validation. In parallel, a partnership was established with a team specializing in web-based educational content to design and implement the intervention on a digital platform.

### Ethical Considerations

This study was approved by the Ethics Committee of the university hospital (approval no. 22.110) on August 12, 2022. All participants provided written informed consent prior to participating.

## Results

To provide a comprehensive description of the *Changeons ensemble* intervention, we followed TIDieR (Template for Intervention Description and Replication) guidelines for reporting complex interventions (Hoffmann et al., 2014). This reporting complements the Intervention Mapping (IM) framework, which guided the intervention development process and structured the presentation of results according to its successive steps (Bartholomew et al., 2016). While IM provided a systematic approach to development of the intervention, TIDieR ensured transparent and detailed documentation of its components to facilitate replication and implementation.

### Step 1: Needs Assessment

#### Literature Review

The literature review identified several factors that can either facilitate or hinder behavior change among older adults with CHD. Because the terminology used to describe these factors varied across studies, it was reorganized according to four commonly defined determinants of behavior change: knowledge, motivation, self-efficacy, and environmental context. Factors with similar conceptual definitions across studies were grouped to ensure consistency and clarity in the analysis.

For each factor, favorable and unfavorable influences reported in the literature were listed. For example, for self-efficacy, favorable influences include previous successful experiences (Jepma et al., 2021) and confidence in one's ability to change (Cleary et al., 2015), whereas unfavorable influences include perceptions of being limited by age or comorbidities (Cleary et al., 2015; Jepma et al., 2021). For motivation, favorable influences include perceiving benefits (Jepma et al., 2021; Young & Barnason, 2014) and having social support (Jepma et al., 2021), while unfavorable influences include low perceived control over the disease (Al-Smadi et al., 2016) and a lack of perceived benefits (Jepma et al., 2021; Young & Barnason, 2014). For knowledge, favorable influences include awareness of the benefits of change (Cleary et al., 2015), whereas unfavorable influences include insufficient information about the disease and information not tailored to individual needs (Young & Barnason, 2014). For environmental context, favorable influences include access to pleasant walking areas (Cleary et al., 2015), while unfavorable influences include lack of time, difficult access to resources, and adverse weather conditions (Cleary et al., 2015; Young & Barnason, 2014).

According to Bartholomew et al. (2016), the importance of each factor was analyzed based on frequency of reporting in the literature, strength of evidence supporting its influence on behavior, and the feasibility of targeting the factor in an intervention. The results of this analysis led to retaining only the three factors considered most relevant and modifiable: knowledge, motivation, and self-efficacy. For each retained factor, strategies to enhance behavior change were identified based on existing evidence. For example, self-efficacy can be targeted through verbal persuasion, modeling, and feedback; motivation can be elicited by exploring perceived benefits and disadvantages and providing professional support; and knowledge can be increased by tailoring information and challenging false beliefs. Environmental context, although relevant, was considered less amenable to modification and thus was not retained as a primary target. This structured analysis ensured that the intervention could focus on the most influential and actionable factors. A table summarizing the process of identifying and analyzing the factors is presented in Supplemental Material 1.

#### Semi-Structured Interviews

In total, 10 people participated in the semi-structured interviews. Of these, six were aged 65 to 74 years, while four were aged 75 to 84 years. Half of the participants had undergone percutaneous coronary intervention (n = 5), and the other half had had coronary bypass surgery (n = 5) within the last year. The majority of the participants were retired (n = 8), while two were employed full-time. Regarding the participants’ physical activity in the first year following their coronary revascularization, two reported having increased their physical activity level, five reported maintaining their prior activities, and three reported not engaging in physical activity.

The results of the qualitative analysis are presented in three main themes related to the themes of the interview guide: the impacts of the health experience on quality of life, behavior change after a coronary revascularization, and the features of the intervention.

#### Theme 1 - The Impacts of Health Experience on Quality of Life

Participants described numerous ways in which CHD affected their daily lives. These impacts encompassed both physical and emotional dimensions, each closely intertwined with their overall quality of life. In this study, quality of life refers to the individual's subjective perception of their health status, emotional well-being, physical and social functioning, pain, and level of energy or fatigue (Maruish, 2011). These experiences revealed a need for better support in understanding and adapting to the physical and emotional consequences of CHD.

#### Impacts on Physical Health status and Daily Functioning

In the first few months following coronary revascularization, many older adults reported symptoms such as pain, shortness of breath, fatigue, and reduced endurance that adversely affects their ability to perform daily and physical activities. These difficulties required significant adaptations, i.e., moderating their physical activities, learning to recognize their limits, and respecting their new pace. Participants recounted the impacts:

“It's my energy [that was impacted]. I lack energy. And the fatigue and pain in my joints and muscles. As I said, if I go to the store and come back with several bags, I really have to think about how I’m going to get them up to my place. […] It's adjustments.” - Participant 06

“[What I found the hardest] is starting over, step by step. You start slowly. You come out of the hospital, you don’t take big steps to start again. I wasn’t the same man anymore.” - Participant 07

Some older adults feel more limited by their health than before the coronary revascularization:

“The worst thing for me is not being able to walk like I did before. Going to dinner on [street name], I liked walking there and walking back. […] I can’t do things like that anymore.” - Participant 01

Others experience a **fear** at having discomfort while engaging in physical activity that is similar to the symptoms they felt during the coronary event:

“We always think about it. Like, for example, when we go cycling, we think how far can we go in case something happens.” - Participant 10

These physical challenges directly influenced participants’ perceived quality of life, altering their energy level, their perception of their health and their physical functioning. These challenges highlight the need for interventions that provide tailored support for physical activity, taking into account the physical impacts of the individual's CHD.

#### Impacts on Emotional Health status

Receiving a CHD diagnosis was often described as a turning point that reshaped how participants perceived themselves and their lives. For some, it provoked shock and disbelief, especially when the diagnosis seemed inconsistent with their healthy lifestyle; for others, it triggered guilt or regret associated with past habits. One participant mentioned:

“It kind of upset me [the diagnosis]. I thought, I’ve always paid a lot of attention to my diet. I exercised and I think I have a good lifestyle and all that, so I thought, it's not worth it.” - Participant 09

For others, this awareness brought about a renewed appreciation of life, encouraging them to live more fully and enjoy meaningful moments:

“I tell myself, we’ll still do the Christmas dinner, the New Year's dinner, maybe it's the last one, you never know what could happen. We enjoy more, we don’t postpone.” - Participant 09

A few participants expressed regrets about the coronary revascularization intervention itself, given the challenge they faced during recovery:

“In my case, it happened so quickly that when I had the surgery, I went back home and I regretted it. I thought, what did I just do?” - Participant 02

Regardless of the emotions experienced, most older adults go through a process of accepting the disease, marked by the ability to put things into perspective and normalize their health condition:

“You have to live with it. […] Maybe it's better to have this than something else. It's not that bad. We get over it, and it's not so bad after all.” - Participant 09

These experiences strongly influence quality of life, shaping their emotional well-being. Recognizing these emotional impacts underscores the need for interventions that acknowledge the emotional dimensions of recovery, in addition to addressing the physical recovery, thereby playing a key role in enhancing overall quality of life following coronary revascularization.

#### Theme 2 – Needs Related to Factors Influencing Behavior Change

The factors influencing behavior change in older adults with CHD were grouped according to the constructs of the Information-Motivation-Behavioral Skills model, i.e., information, motivation, behavioral skills (Fisher et al., 2003), as described below.

*Need for information:* Following coronary revascularization, while some older adults reported feeling sufficiently informed about adopting an active lifestyle, others expressed *ongoing uncertainty about how to safely engage in physical activity and desired more information*. This uncertainty was often related to the type, duration, frequency, and progression of post-revascularization physical activity:

“What exercises should I do? Do I need to move more than once a day? Should I go for walks? Should my walks be just half an hour, just 15 min? Does it need to be 2 h? You know, no one offered me anything, no one explained anything.” - Participant 05

Such lack of information may limit confidence and readiness to adopt or maintain physical activity after revascularization, highlighting the need for individualized guidance.

*Need for motivation:* While some older adults are very motivated to engage in physical activity after coronary revascularization, others are less motivated and need to identify their own sources of motivation. Motivation emerged as a multifactorial construct influenced by both individual and social sources. Among the individual sources of motivation are the *perception of the benefits* of the behavior (e.g., better physical and mental condition, maintaining autonomy and health, a feeling of well-being, more energy and concentration), *self-discipline *(e.g., willpower, developing a habit), *perceived health status* (e.g., desire to recover, seeing progress), and *professional support* (e.g., encouragement). Participants mentioned:

“[Engaging in physical activity allows me to aim for] recovery as quickly as possible to regain my autonomy. Physically and mentally, too. I find that very, very important.” - Participant 02

“Maybe that would be helpful, some kind of encouragement or someone who… You know, with encouragement, you feel more supported. You feel more motivated to do it. Someone who helps you become a bit more aware of it.” – Participant 08

The sources of demotivation include elements related to the *impacts of the disease on physical condition* (e.g., shortness of breath, fatigue, health status, feeling limited compared to others), *aging*, and giving *low priority* to physical activities. One participant mentioned:

“Before, I would climb a small hill and wouldn’t even notice, but now I was (sound of shortness of breath). I found it difficult, and it sapped my motivation a little.” - Participant 05

These findings underscore the importance of supporting older adults as they identify personally meaningful motivators while acknowledging their disease-related limitations.

*Need for confidence in their abilities:* Older adults vary in their level of confidence in their ability to engage in physical activity after a coronary revascularization. Older adults express a need for *support*, clarity in their *life goals*, and noticeable *improvements in their health status* in order to be confident in their ability to engage in physical activity. Participants explained:

“No, [I’m not confident]. Well, because there hasn’t been any improvement. Because I have trouble climbing the stairs. And, um… I feel like I’m at 50% of my capacity.” - Participant 06

“After spending a month in the hospital, you lose a little bit of confidence. Will I feel better tomorrow? I was always out of breath. […] But as the weeks passed, more confidence came back.” - Participant 02

These findings suggest that interventions should support confidence through a gradual progression, personalized goals, and positive reinforcement to help older adults regain a sense of their capability.

#### Theme 3 – Needs Related to the Features of the Intervention

Participants raised various features to be considered when developing online interventions for older adults living with CHD. They have been grouped in terms of the need for a personalized intervention, needs related to professional support, and the need for advice.

**Need for a personalized intervention:** Participants emphasized the importance of an intervention that is tailored to each individual's needs, preferences, and physical condition. This personalization includes enough flexibility to address diverse realities, such as the ability to perform exercises at home or, conversely, a need to participate in group activities. The need for individualized support throughout the process was also highlighted. Participants summarized this idea well:

“I think we need to start with them [the older adults] and see how they feel, what they want to do, and how it's going in the end.” - Participant 09

“What I find helpful in this kind of program is when we appeal to people's intelligence. You know, when they just tell me, ‘Okay, do this,’ it's so-so… But when they explain it, and then the person will do it because they’re convinced, I think that's really, really important in a web program.” - Participant 03

**Needs related to professional support:** Regarding the support needed throughout the intervention, participants expressed a need for validation of their physical activity behaviors, reflections on physical activity, discussions, listening, encouragement, and reassurance. Participants stated:

“Sometimes it just takes a little encouragement from someone outside. I think that would help me. It would have helped me if someone had told me, ‘Okay, 10 min isn’t long, so go do three 10-min walks instead of doing a full 30 min.’” - Participant 08

“[Professional support] might have confirmed more that I was doing the right thing.” - Participant 05

**Need for advice and guidance:** Older adults who have undergone coronary revascularization expressed a desire for concrete advice to help them increase their physical activity. Participants were not only looking for general information, but also for practical guidance on how to begin, how much to do, how to progress safely, and how to adapt their activities based on their symptoms or physical limitations. Such advice was seen as something that could be immediately applied in daily life:

“They could give me one or two tips. Okay, you should do more of this, or do this too, or do less of that at the beginning, you know…” - Participant 05

“Guidance makes it easier because you have a set number of exercises and training sessions, and so much warm-up time. Having clear guidance makes the program easier to follow and more motivating.”- Participant 10

These findings suggest that web-based interventions for older adults with CHD should be personalized to individual abilities and preferences, and provide clear guidance, practical advice, and ongoing professional support to enhance motivation, confidence, and knowledge.

### Step 2: Identification of the Intervention Objectives

The needs assessment led to the identification of intervention objectives. The primary goal of the intervention is to increase physical activity and improve quality of life among older adults living with CHD. To achieve this goal, several factors (i.e., knowledge, motivation, and self-efficacy) were identified that the intervention needs to address. These were formulated as sub-goals: (1) increase knowledge about physical activity and CHD; (2) increase motivation for physical activity; and (3) increase self-efficacy regarding physical activity. These sub-goals were subsequently translated into specific behaviors that participants should engage in by participating in the intervention in order to achieve the main objective. Since quality of life is not a factor of change but rather the overall result of other factors, improvements in knowledge, motivation and self-efficacy are expected to support sustained engagement in physical activity, ultimately contributing to a better quality of life. The objective matrix is presented in Supplemental Material 2.

### Step 3: Intervention Design

The design of the intervention focuses on themes and components, theoretical foundations, behavior change techniques, delivery methods, and the structure of the intervention, with the overarching goal of enhancing physical activity and quality of life among older adults living with CHD.

#### Themes and Components

The themes and components of the intervention were derived from the needs assessment, ensuring that each need identified by older adults was addressed. Table 1 summarizes the key needs and the corresponding intervention themes or components. Some components, such as individualized nursing support, are integrated across all themes to provide continuous guidance throughout the intervention. Overall, the intervention components were designed to be responsive to the expressed needs of participants, providing personalized advice, structured guidance, motivational strategies, and continuous support. By aligning each component with a specific need, the intervention aims to enhance participants’ confidence, motivation, and knowledge regarding physical activity post-revascularization.

**Table 1.** Themes or Components of the Intervention in Line with Identified Needs.

Identified Needs	Themes and Components
Need to acknowledge and adapt to reduced quality of life due to physical and emotional changes	Physical and emotional impacts of CHD Strategies to maintain and improve quality of life
Need for information related to uncertainties about physical activity Need for advice and guidance on how to be more active	Guidance on safe physical activity after coronary revascularization Normal and abnormal signs during physical activity after coronary revascularization Types and intensity of physical activity
Need to identify sources of motivation and motivate self in relation to sources of demotivation	Motivations and barriers Advantages and disadvantages Strategies for self-motivation and for overcoming barriers
Need for support Need for personalized intervention	Individualized nursing support throughout the intervention Peer exchange forum
Need for perceptible improvements in health status	Monitoring progress in an electronic physical activity diary

#### Theoretical Foundations

The Information-Motivation-Behavioral Skills (IMB) model served as the theoretical foundation for this study. The IMB model was chosen due to the convergence of its core constructs (information, motivation, and behavioral skills) with the results of the needs assessment. Additionally, the IMB model complements the IM framework, as both propose needs-based intervention development.

The IMB model posits that an individual with the necessary information and a high level of motivation can apply behavioral skills (i.e., self-efficacy) to achieve behavior change (Fisher et al., 2003). By operationalizing these concepts through the intervention (e.g., providing information to increase knowledge, stimulating motivation by exploring the benefits of change, and fostering self-efficacy through vicarious experience), the intervention addressed the factors influencing behavior change in older adults living with CHD to increase their level of physical activity.

**Figure 1.**
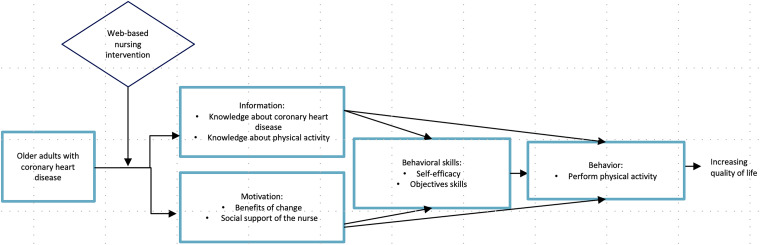
IMB model adapted to the present study (Fisher & Fisher, 1992).

Figure 1, which was adapted with the authors’ permission (Fisher & Fisher, 1992), presents the IMB model and the relationships between the constructs.

#### Behavior Change Techniques

According to Carey et al. (2019), specific behavior change techniques (BCTs) are most effective for influencing each factor of change. For the “knowledge” factor, providing “information about health consequences” is highly impactful (*p* < 0.001). For the “self-efficacy” factor, the selected behavior change techniques include verbal persuasion, prior experiences, emotional states, self-monitoring, vicarious experience, and information on how to perform the behavior. The identified behavior change techniques for the “motivation” factor are exploring the advantages and disadvantages of change, setting change goals, and providing information and professional support. Next, the behavior change techniques were operationalized within the intervention, meaning they were adapted into concrete actions to achieve the desired change. For example, vicarious experience was operationalized through case stories of older adults living with CHD, allowing participants to relate to concrete examples and envision successful change situations. These case stories illustrate the journey of resuming physical activities and improving their quality of life despite the challenges related to CHD, which can help participants become more confident in their ability to do the same and adopt health-promoting behaviors. Table 2 presents the behavior change techniques and their operationalization in the intervention for each of the factors identified above.

**Table 2.** Operationalization of Behavior Change Techniques According to the Factors of Change.

Factors	Behavior Change Techniques	Operationalization
Self-efficacy	Verbal persuasion (Carey et al., 2019)	Nursing support to encourage and reassure participants about their abilities
	Vicarious experience (Carey et al., 2019)	Case story illustrating strategies to resume physical activity and its positive impact on quality of life
	Demonstrating behavior (Carey et al., 2019)	Toolkit with physical activity suggestions and examples
	Self-monitoring of behavior (Carey et al., 2019)	Electronic diary to track physical activity and perceive progress in quality of life
	Reinforcing past and present success (Carey et al., 2019)	Reflexive activities to explore previous and current successes, with reinforcement from the nurse
	Encouraging gradual progress (Carey et al., 2019)	Action plans promoting gradual achievement of objectives Information on the importance of gradual progress
Motivation	Exploring the advantages and disadvantages of change (Carey et al., 2019)	Reflexive activity to identify personal advantages and disadvantages, motivations and obstacles Reflexive activity about the benefits of physical activity on quality of life
	Highlighting strengths and successes (Carey et al., 2019)	Reflexive activity to explore past successes Nursing support to reinforce past achievements and ongoing progress
	Providing information on health consequences (Carey et al., 2019)	Readings and resources explaining the health risks of inactivity and benefits of physical activity
Information	Providing information (Carey et al., 2019)	Advice on physical activity after coronary revascularization Reinforcing one's understanding of how physical activity supports overall health and quality of life Feedback and validation of behaviors

#### Delivery Mode

The delivery mode selected for this project is an individualized asynchronous online intervention, accessible via computer, that includes the support of a nurse. The nurse has clinical experience in behavior change as well as with older adults living with CHD. In this role, the nurse provides personalized feedback that reflects each participant's needs, as well as their level of motivation, knowledge, and self-efficacy regarding physical activity, factors that are key to supporting behavior change (Bartholomew et al., 2016; Kok et al., 2016). Every week, the nurse supports participants through the web-based intervention by highlighting their progress, providing asynchronous written feedback by responding to their answers during reflective activities, offering advice and encouragement, and facilitating discussions and answering questions on the forum. The nurse responds to the participant within 48 h. The intervention is considered asynchronous since the participant can access the intervention at any time and complete the sessions at their convenience. This approach allows older adults to access the intervention at their own pace, accommodating fluctuations in energy and symptoms experienced after coronary revascularization. This mode of delivery also overcomes common barriers to traditional in-person programs, such as transportation challenges or mobility limitations, thereby improving accessibility. The nurse provides technological support to participants when needed, including guidance on platform use and assistance with participation-related difficulties. Step-by-step written instructions are provided for additional support.

#### Structure

Based on the literature (Alley et al., 2020; Lavoie & Dubé, 2022; Wichmann et al., 2020), the intervention consists of seven weekly sessions, each lasting approximately 30 min. With a new session available every week, participants log in with a username and password on a secure Moodle platform (Martin Dougiamas), accessible 24/7. An email reminder is sent to participants after one week of inactivity on the platform. A sequence has been established to maintain logical continuity across all the intervention sessions, as follows: 1) *Introduction*: Present the objectives and content of the session; 2) *Reading*: Provide information to increase the participant's knowledge about physical activity in the context of CHD; 3) *Reflexive activity*: Encourage the participant to reflect on physical activity in the context of CHD and guide them through behavior change on their own; 4) **Action plan**: Set a weekly change goal that the participant wants to achieve; 5) **Conclusion**: Review the content of the session; 6) **Electronic physical activity diary**: Track the participant's progress in their physical activity; 7) **Case story**: Present stories of older adults with similar health experiences; and 8) **Forum**: Exchange ideas with peers and ask questions of the nurse coach. Participants are also given a toolkit, including suggestions for physical activities (e.g., flexibility, balance, resistance, and cardiorespiratory endurance exercises) and the Borg scale of perceived exertion (Borg, 1982).

### Step 4: Production of the Intervention

The seven sessions of the intervention were integrated into the Moodle platform. The visual design of the platform sought to reflect diversity in aging and promotes a positive and joyful view of physical activity (e.g., through images depicting people of different ages being active in a park). Particular attention was paid to accessibility standards to ensure that the intervention can be used comfortably. The writing style emphasized clarity and plain language, and the visual presentation followed readability guidelines regarding font size, color contrast, and layout to support ease of reading and screen navigation (World Wide Web Consortium, 2025). Table 3 presents the titles of each session of the intervention and a summary of their content, and illustrative examples showing some components of the intervention are shown in Supplemental Material 3.

**Table 3.** Summary of the Intervention Sessions’ Content.

Session	Content	
1. Let's Get to Know Each Other	Readings	Information on coronary artery disease, its causes, and treatments
	Reflective activities	Getting to know each other Portrait of the health experience
2. Adapting to Your New Reality	Readings	Types and intensity of physical activity Recommendations related to physical activity after a coronary event Normal and abnormal signs during physical activity
	Reflective activities	Role of physical activity in my life Past experiences of change
3. I can be more active!	Readings	Advantages and disadvantages of physical activity Motivations and barriers to physical activity
	Reflective activities	Your advantages and disadvantages Your motivations and barriers
4. I’m making progress!	Readings	Achieving your goals
5. I maintain the change	Readings	Strategies to maintain motivation
	Reflective activities	Staying motivated
6. I overcome relapse	Readings	Relapses – Are they normal? What to do during a relapse
	Reflective activities	Anticipating high-risk relapse situations
7. My learning and next steps	Reflective activities	Review of my learnings

## Discussion

This article describes the development of *Changeons ensemble*, a web-based nursing intervention aimed at promoting physical activity and quality of life in older adults with CHD. In collaboration with a multidisciplinary team of experts, seven sessions were developed. Using the IM framework (Bartholomew et al., 2016), the intervention was systematically developed, ensuring that it aligns with both empirical evidence and the needs of the target population. Specifically, its development was informed by the Information-Motivation-Behavioral Skills model (Fisher et al., 2003) and a comprehensive needs assessment that combined a literature review and semi-structured interviews with older adults. This theory-informed approach, which integrates evidence-based behavior change techniques, may increase the likelihood of sustained behavior change by directly targeting modifiable factors (i.e., information, motivation, behavioral skills), thereby enhancing the relevance and usability of the intervention for older adults (Gilchrist et al., 2024). Electronic diary to track physical activity may enhance self-monitoring and awareness of physical activity behaviors, which may in turn support self-efficacy (Carey et al., 2019). Case stories may provide social modeling and increase perceived capability by allowing participants to identify with peers who have successfully engaged in physical activity. Reflective activities and action planning may further facilitate goal setting and reinforce intrinsic motivation (Carey et al., 2019). Together, these mechanisms are intended to support improvements in physical activity behavior and, ultimately, quality of life. Although older adults benefit from physical activity (Anderson et al., 2016; Lahtinen et al., 2018; Lopes et al., 2020), few studies have examined how interventions aimed at increasing physical activity actually influence quality of life outcomes (Raafs et al., 2020; Taylor et al., 2021). Future studies could further explore which components of the intervention contribute most to improvements in quality of life.

One of the main contributions of this study lies in the integration of older adults’ perspectives throughout the development process. The needs assessment highlighted facilitators and barriers to physical activity after coronary revascularization. Factors such as lack of knowledge, variable motivation, low self-efficacy, and the physical and emotional impacts of CHD emerged as particularly salient and were addressed through targeted intervention components. By adopting a participatory planning approach, the intervention was further refined to reflect the lived experiences and preferences of older adults living with CHD, enhancing its acceptability, potential impact, and relevance for improving quality of life**.** Interventions that adopt participatory approaches have been shown to be more effective because they reflect the needs, preferences, and lived contexts of the people they are designed to support, thereby enhancing acceptability, and real-world impact (Constantin et al., 2022).

A key innovation of the *Changeons ensemble* intervention is the inclusion of individualized nursing support, a feature that remains underexplored in the context of web-based interventions. This support appears essential for facilitating behavior change and maintaining quality of life in an aging population (Beishuizen et al., 2019; van Middelaar et al., 2018), as it enables personalization based on individual needs. For older adults with CHD, behavior change can involve navigating multiple comorbidities, physical limitations, and different rehabilitation goals, making standardized approaches less effective (Ambrosetti et al., 2020). While some researchers advocate for such support in web-based interventions (Su et al., 2025), its specific impact on the lifestyle habits of older adults remains under-evaluated, a gap our forthcoming study (Lavoie & Dubé, 2025) aims to address.

The results of this study are consistent with previous research emphasizing the need for personalized, flexible, and supportive interventions for older adults (Happe et al., 2021; Lavoie & Dubé, 2022; Wilson et al., 2021). Studies among older adult and individuals living with CHD have shown that digital health interventions can be effective in promoting physical activity when they incorporate elements such as tailored recommendations, interactive features, and professional support (Agyei et al., 2024; Lavoie & Dubé, 2022; Walha et al., 2025). Our findings show that older adults value interventions that provide not only educational content but also encouragement, validation, and individualized guidance. The needs assessment revealed that older adults with CHD often experience reduced quality of life, manifested by significant emotional and physical impacts and highlighting a clear need for tailored support. This finding aligns with similar observations in broader adult populations (Douma et al., 2024; Gray et al., 2022; Yang et al., 2025).

A notable strength of this study is the rigorous and transparent methodology used to develop the intervention, along with comprehensive documentation and a detailed description of the development process. The use of the IM framework enabled a structured and iterative process that incorporated scientific evidence, theoretical models, and experiential knowledge. Additionally, the inclusion of a participatory planning group enriched the intervention by incorporating diverse perspectives from both healthcare professionals and older adult with lived experience. The detailed description of the intervention development process and its components could guide the development of future digital health interventions aimed at promoting health among older adults, or even adapt it to other clinical contexts or populations. Moving forward, research should focus on assessing the intervention's effects in terms of changing physical activity behavior and improving quality of life, as well as its feasibility and acceptability in real-world clinical settings. Particular attention should be paid to understanding how each component of the intervention, such as individualized nursing support, contributes to outcomes. Ultimately, by addressing key behavioral factors and embedding professional support in a user-centered digital format, *Changeons ensemble* offers a promising avenue to support lifestyle change and secondary prevention among older adults with CHD.

Some limitations should be acknowledged. First, although the needs assessment provided valuable insights, the sample size for the semi-structured interviews was relatively small (n = 10), which may limit the transferability of the findings. Given that this needs assessment was conducted in a single regional context, the findings may not be fully transferable to older adults living with CHD in other healthcare systems or clinical contexts. Future studies should include a larger and more diverse sample to provide a broader perspective on older adults’ needs and preferences and to enhance transferability.

The absence of a formal assessment of thematic saturation may have affected the completeness of the identified needs; however, the purpose of this study was exploratory and focused on informing intervention development. Second, the participatory planning group was primarily composed of health professionals, with only one patient partner. This may have resulted in a predominantly clinical perspective influencing intervention design decisions. Future work should involve a greater representation of older adults with CHD to better capture a diversity of lived experiences in the co-design process (Grindell et al., 2022; Slattery et al., 2020). As this study focused on a web-based intervention development, considerations related to digital literacy, access to technology, and individual preferences may have influenced participation and engagement in the co-design process. These factors should be considered in future studies examining the acceptability and feasibility of the intervention in broader populations of older adults. Finally, the intervention has yet to be tested in a real-world setting. Future research should therefore focus on a feasibility and usability study to assess its acceptability, and preliminary effects on physical activity behaviors and quality of life among older adults living with CHD. Studies should also explore participants’ perceived benefits and experiences of using the intervention in order to better understand its impact from the users’ perspective. In addition, future implementation efforts may benefit from establishing partnerships with healthcare organizations or community-based services to support the delivery of the intervention in real-world settings.

## Conclusion

This study provides a detailed description of the development process of *Changeons ensemble*, a web-based intervention to promote physical activity and quality of life among individuals living with CHD. Based on a rigorous methodology and a needs-tailored approach, this intervention could inform the development of future digital health initiatives for older adults. However, further research is needed to evaluate its acceptability, feasibility, and effectiveness in real-world settings. This study contributes to the growing body of knowledge on digital health interventions for older adults and underscores the importance of user-centered design in promoting healthy aging. With aging populations and the rise in chronic diseases, it is essential to continue exploring and developing innovative and accessible strategies for supporting the health and well-being of older adults. As digital technologies become central to healthcare, interventions must be accessible and tailored to older adults’ diverse needs. Efforts must be made to overcome technological barriers and ensure effective implementation within healthcare services.

## Supplemental Material

sj-docx-1-cjn-10.1177_08445621261452849 - Supplemental material for Supporting Physical Activity and Quality of Life in Older Adults with Coronary Heart Disease: Development of a Web-Based Nursing Intervention Supplemental material, sj-docx-1-cjn-10.1177_08445621261452849 for Supporting Physical Activity and Quality of Life in Older Adults with Coronary Heart Disease: Development of a Web-Based Nursing Intervention by Audrey Lavoie and Véronique Dubé in Canadian Journal of Nursing Research

sj-docx-2-cjn-10.1177_08445621261452849 - Supplemental material for Supporting Physical Activity and Quality of Life in Older Adults with Coronary Heart Disease: Development of a Web-Based Nursing Intervention Supplemental material, sj-docx-2-cjn-10.1177_08445621261452849 for Supporting Physical Activity and Quality of Life in Older Adults with Coronary Heart Disease: Development of a Web-Based Nursing Intervention by Audrey Lavoie and Véronique Dubé in Canadian Journal of Nursing Research

sj-docx-3-cjn-10.1177_08445621261452849 - Supplemental material for Supporting Physical Activity and Quality of Life in Older Adults with Coronary Heart Disease: Development of a Web-Based Nursing Intervention Supplemental material, sj-docx-3-cjn-10.1177_08445621261452849 for Supporting Physical Activity and Quality of Life in Older Adults with Coronary Heart Disease: Development of a Web-Based Nursing Intervention by Audrey Lavoie and Véronique Dubé in Canadian Journal of Nursing Research
