# Supplementary material for: Supporting Physical Activity and Quality of Life in Older Adults with Coronary Heart Disease: Development of a Web-Based Nursing Intervention
Source: Can J Nurs Res. 2026 May 26;58(3):329–43. doi: 10.1177/08445621261452849 (PMC13454406; doi:10.1177/08445621261452849)
Supplement: Supplementary file 1 [file sj-docx-1-cjn-10.1177_08445621261452849.docx]

**Supplemental Material 1.**  Factors likely to contribute to behavior change in older adults with CHD

| **Factors identified in the literature** | **Importance** | **Potential for change** |
| --- | --- | --- |
| **Self-efficacy:** Belief in one’s ability to change a behavior (Bandura, 2018; Fisher et al., 2003) | | |
| **Favorable factors:**   - Previous successful experience of change (Jepma et al., 2021) - Control over one’s ability to change, confidence in one’s capacity to change (Cleary et al., 2015)   **Unfavorable factors:**   - Perception of being limited by one’s condition, comorbidities, functional limitations (Cleary et al., 2015; Jepma et al., 2021), and age (Cleary et al., 2015) - Perception of being too old to change (Young & Barnason, 2014) - Previous experience of failure in change efforts (Young & Barnason, 2014) - Required skills for change (Jepma et al., 2021) | +++ | +++  Modifiable by acting on verbal persuasion, past experiences, emotional states, vicarious experience, and information on how to perform the behavior (Bandura, 2018; Carey et al., 2019) |
| **Environmental context**: Aspects of the environment that influence behavior change (Carey et al., 2019) | | |
| **Favorable factors:**   - Indoor environment conducive to walking (Cleary et al., 2015) - Pleasant outdoor environment (Cleary et al., 2015)   **Unfavorable factors:**   - Lack of time (Cleary et al., 2015) - Difficulty accessing resources, including cardiac rehabilitation (Cleary et al., 2015; Young & Barnason, 2014) - Weather conditions (e.g., rain, snow, cold or excessive heat) (Cleary et al., 2015) | ++ | +  Hardly modifiable. Possibility of modification through problem-solving (e.g., identification of alternative physical activities) (Carey et al., 2019) |
|  |  |  |
|  |  |  |
| **Motivation:** Attitudes towards change, perceived benefits, disadvantages, and social support (Fisher et al., 2003) | | |
| **Favorable factors:**   - Perception of the benefits of change (Jepma et al., 2021; Young & Barnason, 2014) - Perception of the threat related to the behavior to be changed (Jepma et al., 2021) - Health improvement (Cleary et al., 2015) - Sense of well-being and enjoyment (Cleary et al., 2015) - Desire to return to pre-surgery activities (Cleary et al., 2015) - Having an exercicse partner (Cleary et al., 2015) - Support from family and professionals (Jepma et al., 2021)   **Undefavorable factors:**   - Lack of motivation (Cleary et al., 2015) - Perception of few consequences or low control over the disease (Al-Smadi et al., 2016) - Perception of few benefits from change (Jepma, 2021; Young et Barnason, 2014) - Perception of being cured of the disease following surgery (Young & Barnason, 2014) | +++ | +++  Elicit motivations by exploring the benefits and disadvantages, identify change goals, and provide information and professional support (Carey et al., 2019) |
| **Knowledge**: Acquisition of health information (Fisher et al., 2014; Rincón Uribe et al., 2021) | | |
| **Favorable factors:**   - Knowing the benefits of change (Cleary et al., 2015)   **Undefavorable factors:**   - Lack of knowledge about the disease and secondary prevention (Young & Barnason, 2014) - Information that does not meet individual needs (Young & Barnason, 2014) | **++** | **+++**  Increase knowledge by providing information (Carey et al., 2019). Challenging false beliefs (Fisher et al., 2014; Rincón Uribe et al., 2021) |

Importance = The significance of the factor in relation to behavior change, based on how often it is mentioned in the literature;

Potential for change = The possibility of acting to modify this factor through an intervention; + = of low importance or difficult to modify;

++ = of moderate importance and somewhat modifiable; +++ = very important and modifiable

References

Al-Smadi, A. M., Ashour, A., Hweidi, I., Gharaibeh, B., & Fitzsimons, D. (2016). Illness perception in patients with coronary artery disease: A systematic review. *International Journal of Nursing Practice*, *22*(6), 633-648. <https://doi.org/https://doi.org/10.1111/ijn.12494>

Bandura, A. (2018). *Auto-efficacité : Le sentiment d'efficacité personnelle* (D. B. Université, Ed. 3 ed.). de Boeck Université.

Carey, R. N., Connell, L. E., Johnston, M., Rothman, A. J., de Bruin, M., Kelly, M. P., & Michie, S. (2019). Behavior Change Techniques and Their Mechanisms of Action: A Synthesis of Links Described in Published Intervention Literature. *Annals of Behavioral Medicine*, *53*(8), 693-707. <https://doi.org/10.1093/abm/kay078>

Cleary, K., LaPier, T., & Rippee, A. (2015). Perceptions of exercise and quality of life in older patients in the United States during the first year following coronary artery bypass surgery. *Physiotherapy Theory and Practice*, *31*(5), 337-346. <https://doi.org/10.3109/09593985.2015.1004770>

Fisher, W. A., Fisher, J. D., & Shuper, P. A. (2014). Social psychology and the fight against AIDS: An Information–Motivation–Behavioral Skills Model for the prediction and promotion of health behavior change. In J. M. Olson & M. P. Zanna (Eds.), *Advances in Experimental Social Psychology* (Vol. 50, pp. 105-193). Academic Press. <https://doi.org/https://doi.org/10.1016/B978-0-12-800284-1.00003-5>

Jepma, P., Snaterse, M., Du Puy, S., Peters, R. J. G., & op Reimer, W. J. M. S. (2021). Older patients’ perspectives toward lifestyle-related secondary cardiovascular prevention after a hospital admission—a qualitative study. *Age and Ageing*, *50*(3), 936-943. <https://doi.org/10.1093/ageing/afaa283>

Rincón Uribe, F. A., Godinho, R. C. d. S., Machado, M. A. S., Oliveira, K. R. d. S. G., Neira Espejo, C. A., de Sousa, N. C. V., . . . Pedroso, J. d. S. (2021). Health knowledge, health behaviors and attitudes during pandemic emergencies: A systematic review. *PloS one*, *16*(9), e0256731-e0256731. <https://doi.org/10.1371/journal.pone.0256731>

Young, L., & Barnason, S. (2014). Older patients’ perception and experience with lifestyle changes following cardiac revascularization. *American Journal of Clinical Medicine*, *10*(1), 30-38. <http://aapsus.org/wp-content/uploads/Older-Patients-Perception.pdf>
