# Supplementary material for: Supporting Physical Activity and Quality of Life in Older Adults with Coronary Heart Disease: Development of a Web-Based Nursing Intervention
Source: Can J Nurs Res. 2026 May 26;58(3):329–43. doi: 10.1177/08445621261452849 (PMC13454406; doi:10.1177/08445621261452849)
Supplement: Supplementary file 2 [file sj-docx-2-cjn-10.1177_08445621261452849.docx]

**Supplemental Material 2**. Objective matrix of the *Changeons ensemble* intervention

| **At the end of the intervention, older adults with CHD will have increased their level of physical activity improved their quality of life*** | **Change objectives** | | |
| --- | --- | --- | --- |
|  | Factors likely to contribute to behavior change in older adults living with coronary heart disease | | |
|  | Knowledge | Motivation | Self-efficacy |
|  | The intervention will enable older adults with CHD to... | | |
|  | **Increase their knowledge about physical activity and CHD** | **Increase motivation to engage in physical activity** | **Increase self-efficacy in physical activity** |
|  | - Know about coronary artery disease and its treatment - Identify the health benefits of physical activity - Understand the consequences of physical inactivity on health - List different ways of being more active on a daily basis - Identify normal and abnormal signs of physical activity - List recommendations for physical activity following bypass surgery and/or percutaneous coronary intervention - Describe the different types and intensities of physical activity - Explain the process of lifestyle change | - Identify the benefits of physical activity in your life - Identify motivations for physical activity - Express motivation for physical activity | - Express confidence in physical activity abilities - Demonstrate physical activity skills - Feel confident in their abilities by observing their peers - Perceive few barriers to physical activity - Recognize past successes - Recognize current successes - Identify strategies to overcome barriers to physical activity |

*Improvements in knowledge, motivation, and self-efficacy are expected to contribute to enhanced physical activity levels and, ultimately, to better quality of life.
