# Supplementary material for: Supporting Physical Activity and Quality of Life in Older Adults with Coronary Heart Disease: Development of a Web-Based Nursing Intervention
Source: Can J Nurs Res. 2026 May 26;58(3):329–43. doi: 10.1177/08445621261452849 (PMC13454406; doi:10.1177/08445621261452849)
Supplement: Supplementary file 3 [file sj-docx-3-cjn-10.1177_08445621261452849.docx]

**Supplemental Material 3.** *Changeons ensemble* intervention

***Illustration***

**Electronic physical activity diary**	**Case story**

**Borg scale	Toolkit with physical activity suggestions**

**Reflexive activity	Action plan**
